# The Multifactorial Memory Questionnaire and Quality of Life: A Longitudinal Study in Parkinson’s Disease

**DOI:** 10.3390/brainsci15010066

**Published:** 2025-01-13

**Authors:** Emily J. Corti, Natalie Gasson, Hayley Grant, Brayden Wisniewski, Andrea M. Loftus

**Affiliations:** 1 School of Population Health, Curtin University, GPO Box U1987, Bentley, WA 6102, Australia; 2 Curtin Neuroscience Research Laboratory, Curtin University, GPO Box U1987, Bentley, WA 6102, Australia

**Keywords:** Parkinson’s disease, metamemory, quality of life

## Abstract

Background/Objectives: Objective memory decline is associated with poor quality of life (QOL) in Parkinson’s disease (PD, but it is unclear what role perception of memory (metamemory) plays. The Multifactorial Memory Questionnaire (MMQ) measures metamemory and is proposed to have a three-factor structure, but the factor structure of the MMQ in PD has not been explored. The current study examined (i) the factor structure of the MMQ in PD and (ii) the relationship between the metamemory and QOL in PD. Methods: This longitudinal, observational study involved 149 participants with PD (98 males, M age = 65.78 years, SD = 9.25). Participants completed the MMQ and the Unified Parkinson’s Disease Rating Scale (disease severity) at baseline, and the Parkinson’s Disease Questionnaire-39 (QOL) two years later. Results: Confirmatory factor analysis revealed both the three-factor and four-factor models were inadequate. Exploratory factor analysis resulted in a four-factor solution. The contentment and ability subscales from the original factor structure remained, while the strategies subscale was divided into external strategies and internal strategies. Disease severity, ability, and internal strategies uniquely predicted QOL. Individuals who reported low subjective memory ability and high use of internal strategies reported worse QOL two years later. Conclusions: These findings indicate that metamemory predicts QOL in PD and suggest that improved perceived memory ability and strategy use could offer avenues for improved QOL.

## 1. Introduction

Parkinson’s disease (PD) is a neurodegenerative disorder characterized by impairments in motor and cognitive functioning [1] that significantly impact an individual’s quality of life (QOL; [2,3,4]). Measurement of QOL is important, as it indicates the impact of PD symptoms on everyday life, and reduced QOL is associated with negative self-appraisal of symptoms [5]. Identification and understanding of the determinants of QOL in PD may assist in the development of management strategies to alleviate the negative impacts of PD.

Metamemory is one aspect of cognition that may contribute to QOL in those with PD. Metamemory refers to an individual’s subjective perception of their memory abilities and comprises three components: contentment, ability, and strategy [6]. Contentment refers to self-reported feelings about one’s memory performance, ability refers to self-reported memory mistakes, and strategy refers to self-reported memory strategy use [6]. Metamemory predicts QOL in healthy adults and clinical populations, including PD, mild cognitive impairment (MCI), and Multiple Sclerosis [5,6,7,8]. People with PD frequently report memory problems and often perceive their own memory to be poor [9,10]. Prakash et al. [11] examined the impact of non-motor symptoms, including self-reported attention/memory, on QOL in those with PD. At the two-year follow-up point, self-reported memory problems were one of the strongest predictors of poor QOL. Although this study did not use the Multifactorial Memory Questionnaire (MMQ, [12]) as a measure of metamemory, the subjective, self-reported memory questionnaire required participants to judge their perceptions of memory and could, therefore, be considered a measure of metamemory. Jenny et al. [13] reported a significant relationship between subjective memory complaints and the QOL domain regarding cognition in PD, whereby increased memory complaints were associated with poor cognition-related QOL. Although people with PD report memory problems, it is unclear whether their subjective perceived memory performance corresponds with objective memory performance [10,14]. A systematic review reported that three large PD studies demonstrated a significant relationship between subjective and objective memory performance, whereby those with PD who perceived their memory to be poor also scored poorly on objective tests of memory [15]. However, four PD studies reported no relationship between subjective and objective memory performance, suggesting that people with PD may perceive their memory to be poor regardless of their objective memory performance (i.e., impaired metamemory, [15]). It therefore remains unclear whether people with PD are able to accurately report their actual memory functioning.

Montejo et al. [8] investigated the relationship between self-reported memory and domain-specific QOL in an older adult population. Self-reported memory complaints (i.e., memory contentment) significantly correlated with both global and domain-specific QOL, whereby increased complaints were associated with decreased QOL. Domain-specific QOL included activities of daily living, health status, emotional problems, physical activity and fitness, and social support [8]. These findings were supported by [16], which reported that subjective cognitive impairment (including memory) in older adults was significantly and negatively associated with specific QOL domains, including mobility and social functioning. This suggests that negative perceptions of memory ability are associated with poor QOL in older adults and supports the notion that perception of memory (i.e., metamemory) plays an important role in determining QOL in non-clinical populations.

In Multiple Sclerosis (MS), metamemory contentment and ability are significantly correlated with QOL, such that higher self-reported contentment and memory ability are associated with higher QOL [6]. Metamemory contentment significantly predicts QOL in people with MS, beyond that predicted by depression and physical limitations. For those with mild cognitive impairment (MCI), metamemory negatively impacts QOL, and this relationship is stronger for people with MCI than those without [5]. Maki et al. [5] suggest that people with MCI are aware of their cognitive deficits and subsequently negatively evaluate their memory function. This is also evident in PD. Self-rated changes in memory performance across time were worse in people with PD compared to age- and gender-matched controls, suggesting that those with PD have intact awareness of cognitive decline [17]. Considering that MCI is common in people with PD [18] and that people with PD are aware of cognitive deficits, it seems reasonable to suggest that a similar relationship may exist for metamemory and QOL. If metamemory predicts QOL in PD, metamemory interventions may be used to improve QOL.

Evidence for a relationship between metamemory and QOL in PD is inconclusive. While some research has suggested that poor metamemory is predictive of QOL in PD [11,19], there is also evidence that metamemory is predictive only of specific aspects of QOL, and that there is no association between metamemory and overall QOL [13]. One possible explanation for the mixed findings may relate to the different measures of metamemory/self-reported memory used. Although originally developed with healthy older adults [12], the three-factor MMQ has been validated across diverse settings and populations, including those with [20,21] and without memory issues [21,22,23]. A systematic review and meta-analysis reported that the MMQ demonstrates consistent reliability and validity across studies, regardless of language, population, or study design [24]. However, some studies have indicated that a four-factor solution to the MMQ, with the strategy subscale divided into internal strategies and external strategies, is more appropriate than the three-factor one [21,25,26]. It has been suggested that the four-factor solution should be further evaluated [24], as it may provide more information about strategy use and may be used to develop strategy-based interventions to improve QOL and disease management [27]. As the MMQ has not been validated in an English-speaking PD population, it is currently unknown whether the three-factor solution to the MMQ is appropriate for capturing metamemory in PD and what aspects of metamemory are important to QOL in PD. The present study had two key aims. The first was to examine the factor structure of the MMQ in a large, Australian community-based PD cohort. The second was to determine whether scores on the MMQ would predict QOL 2 years later. To achieve this, data for the MMQ were collected at time 1 (study entry), and QOL data were collected at time 2 (2 years later). This design allowed us to explore the longitudinal impact of metamemory on QOL in those with PD.

## 2. Materials and Methods

### 2.1. Participants

Individuals with idiopathic PD from Western Australia were invited to participate in a larger ParkC project (www.parkc.co, accessed on 9 January 2025) through general advertising, support groups, and community presentations. Participants were included if they had a formal diagnosis of PD and if they scored above 24 on the Mini Mental State Examination (MMSE, [28]) at study entry, suggesting no global cognitive impairment was present. This study was approved by a university ethics committee, and all research was conducted in accordance with the Declaration of Helsinki. All participants provided written informed consent.

### 2.2. Power Analysis

For the exploratory factor analysis, research suggests that an approximately 5:1 ratio of participants to items provides an adequate sample size [29]. For the regression analysis, assuming a three-factor model for the MMQ, the results of an a priori power analysis suggested that a minimum sample size of 77 was required to achieve a medium effect size at the desired power level of 0.80 and for a statistical significance level of 0.05 [30]. Sample characteristics at Time 1 can be found in Table 1.

### 2.3. Measures

Demographics. A study-specific demographic questionnaire recorded age, gender, and PD information.

Disease Severity. The Hoehn and Yahr scale (H&Y) was administered as part of the Unified Parkinson’s Disease Rating Scale-III (UPDRS–III) by a trained assessor and provided a staging score indicative of disease severity. The H&Y is a one-item descriptive scale that estimates PD symptom severity based on clinical and functional disability [31]. Ratings range from 1–5 as follows: unilateral presentation (1); bilateral presentation without balance difficulties (2); bilateral presentation with presence of postural instability, physically independent (3); severe disability, still able to walk or stand unassisted (4); and wheelchair-/bed-bound, unless aided (5).

Metamemory. The Multifactorial Memory Questionnaire (MMQ, [12]) measures three aspects of metamemory: contentment, ability, and strategy. This self-report questionnaire requires participants to respond to statements about their memory over the past two weeks using a five-point Likert scale. The contentment subscale comprises 18 items which are summed to give an MMQ-contentment score, with higher scores indicating greater memory contentment (ranging 0–72). The ability subscale comprises 20 items, which are summed to give an MMQ-ability score, with higher scores indicating greater self-reported memory ability (ranging 0–80). The strategy subscale contains 19 items, which are summed to give an MMQ-strategy score, with higher scores indicating greater self-reported memory strategy use (ranging 0–76). Troyer and Rich [12] reported that the MMQ demonstrated good content validity, factorial validity, test–retest reliability (r = 0.86–0.93), and construct validity, both convergent (r = 0.27–0.70) and discriminant. The researchers also found good internal consistency (α = 0.83–0.95). A principal component analysis of metamemory yielded three factors, including contentment, ability, and strategy [12]. Data for the MMQ part of this study were collected at time 1 (baseline).

Quality of life. The Parkinson’s Disease Questionnaire-39 (PDQ-39; [32]) is a PD-specific measure of QOL over a 1-month period. The PDQ-39 covers 8 QOL subscales, including mobility (10 items), activities of daily living (6 items), emotional well-being (6 items), stigma (4 items), social support (3 items), cognition (4 items), communication (3 items), and bodily discomfort (3 items). Participants responded to questions using a five-point Likert scale (never (0)–always/cannot do at all (4)). A score for each dimension was calculated by summing the question scores for each domain, then dividing this by a multiple of four and the number of questions in that domain. This was multiplied by 100 to yield the scale score. A single index score was used in the present study and was calculated by summing the eight scale scores and dividing this by eight. Scores ranged from 0 to 100, with higher scores indicating lower QOL. The PDQ-39 was shown to be internally consistent for all scales except for social support, which was only slightly below the desired alpha level (α = 0.70; [33]). The measure also demonstrated good content validity [33]. Data for the QOL component of this study were collected at time 2 (two years later).

### 2.4. Procedure

After contacting ParkC, potential participants were sent an information package and re-contacted ParkC if they decided to participate. A ParkC employee then scheduled an assessment and sent out a questionnaire pack to be completed at home. The questionnaire pack included information about the study, the MMQ, the PDQ-39, and additional questionnaires relevant to the overarching study (a longitudinal project tracking PD symptoms every two years over a ten-year period). At the testing session, the trained assessor ensured the participant had read the information sheet, answered any questions they had, and asked the participant to read and sign the consent form. Participants completed the MMSE, followed by a series of cognitive assessments and the UPDRS. Sessions lasted approximately 2–2.5 h, and participants were provided with regular breaks. Participants were thanked for their time and asked if they would like to return for time 2 testing in two years’ time. Participants repeated the above procedure two years later.

### 2.5. Statistical Analysis

Data were entered into IBM SPSS version 29. Participants who did not have substantially complete data (missing more than 10% on any measure) at two time points (separated by two years) were omitted from analysis. For scales with less than 10% of data missing, mean substitution was used to replace missing values. A maximum-likelihood confirmatory factor analysis was conducted using IBM SPSS AMOS version 29 for the three-factor and four-factor models of the MMQ. The three-factor model was based on the original factor structure reported by Troyer and Rich [12]. The four-factor model was based on the items reported by Fort et al. [25]. Two items (C9, S3) that did not load in the Fort et al. [25] study were included in the model based on theoretical assumptions. A parallel analysis determined the number of factors for an exploratory factor analysis (EFA). Parallel analysis was conducted in SPSS version 29 using O’Connor [34] syntax for parallel analysis. Based on the results of the parallel analysis, an EFA with a four-factor forced solution was then conducted using principal axis factoring with an oblique rotation. Bivariate correlations and Hierarchical Multiple Regression were conducted using IBM SPSS version 29. Assumption testing indicated that assumptions of multicollinearity, normality, linearity, and homoscedasticity of residuals had been met.

## 3. Results

### 3.1. Confirmatory Factor Analysis

A maximum-likelihood confirmatory factor analysis was conducted for both the three-factor and four-factor models of the MMQ. The three-factor model did not yield adequate fit: χ^2^ = 2674.50, df = 1536, *p* < 0.001, χ^2^/df = 1.74, CFI = 0.737, RMSEA = 0.071, SRMR = 0.083. The overall fit of the four-factor model was also inadequate: χ^2^ = 2610.16, df = 1533, *p* < 0.001, χ^2^/df = 1.70, CFI = 0.751 RMSEA = 0.069, SRMR = 0.082, although the four-factor was a significantly better fit than the three-factor model Δχ^2^ = 64.34, df_diff_ = 3, *p* < 0.001.

### 3.2. Exploratory Factor Analysis

**Figure 1:**
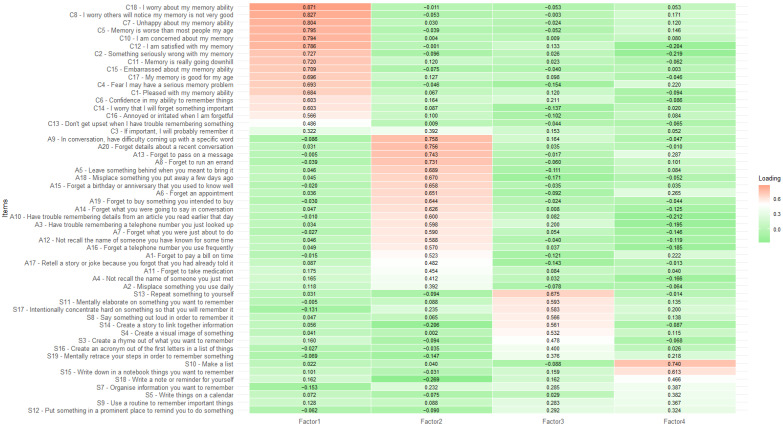
Heatmap of the promax-rotated factor structure of the Multifactorial Memory Questionnaire. Note. Factor 1 = contentment, Factor 2 = ability, Factor 3 = internal strategies, Factor 4 = external strategies. “A” denotes ability items, “C” denotes contentment Items, and S denotes strategies items.

Bartlett’s test of sphericity suggested that the data were suitable for factor analysis (χ^2^ = 5124.31, df = 1596, *p* < 0.001). A Kaiser–Meyer–Olkin value of 0.857 also indicated good factorability of the data. A parallel analysis indicated that four factors should be retained, which was supported by the scree plot. An EFA with a four-factor forced solution was then conducted. The four factors combined accounted for 46.13% of total variance in MMQ scores. Items with a factor loading of >0.30 were included in the respective factors. The factor names proposed by Fort et al. [25] were applied to the current factors. Factor loadings for each item are reported in Figure 1. Factor one was named contentment and contained all but 1 (item 9) of the 18 items in the original contentment subscale. The contentment subscale produced an eigenvalue of 16.38, accounting for 28.74% of variance in MMQ. Factor two was named ability and contained all 20 of the items in the original ability subscale. The ability subscale produced an eigenvalue of 4.56, accounting for 8.00% of the variance in MMQ. Factor three was named internal strategies and contained nine items from the original strategy subscale. The internal strategies subscale produced an eigenvalue of 3.10, accounting for 5.43% of the variance in MMQ. Factor four was named external strategies and contained seven items from the original strategy subscale. The external strategies subscale produced an eigenvalue of 2.25, accounting for 3.95% of the variance in MMQ. Factors three and four contained all but 3 (items 1, 2, and 6) of the 19 items in the original strategy subscale.

Examination of the factor loadings in the pattern matrix revealed that 4 of the 57 items should be excluded due to unsuitability. Contentment item nine (“When I have trouble remembering something, I’m not too hard on myself”.) from the original questionnaire did not load sufficiently (>0.30) onto any factor and was removed. Strategies item one (“Use a timer or alarm to remind you when to do something”.), item two (“Ask someone to help you remember something or to remind you to do something”.), and item six (“Go through the alphabet one letter at a time to see if it sparks a memory for a name or word”.) also did not load sufficiently onto any factor. The pattern matrix for the exploratory factor analysis is presented in Figure 1. Contentment item three (“If something is important, I will probably remember it.) loaded onto both the contentment and ability subscales. Item three was retained in the contentment subscale based on theoretical assumptions.

### 3.3. Reliability

**Figure 2:**
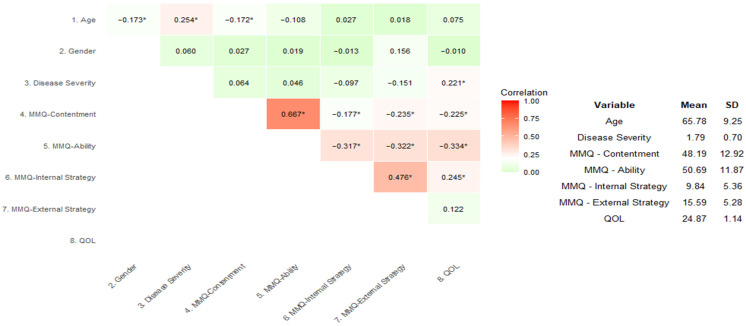
Means, standard deviations, and heatmap of bivariate correlations (Pearson’s r) between variables. Note. MMQ = Multifactorial Memory Questionnaire (metamemory). QOL = quality of life; higher scores indicate poorer quality of life. * *p* < 0.05.

The internal consistency for each factor was calculated using Cronbach’s alpha. The internal consistencies of all scales were sound and exceeded the minimum 0.70 threshold [35]. Cronbach’s alpha for the contentment subscale was 0.946, that for the ability subscale was 0.931, that for the internal strategies subscale was 0.801, and that for the external strategies subscale was 0.744. Bivariate correlations examined relationships between the criterion, predictor, and control variables. Age and gender did not significantly correlate with QOL and were omitted from subsequent analyses. MMQ-external strategy did not correlate with QOL and was also omitted from subsequent analyses. Bivariate correlations are presented in Figure 2.

### 3.4. Hierarchical Multiple Regression Analysis

At step one of the HMRA, disease severity accounted for a significant 4.9% of the variance in QOL, R^2^ = 0.049, F (1, 147) = 7.56, *p* = 0.007. At step two, MMQ-contentment, MMQ-ability, and MMQ-internal strategy were included and accounted for an additional 14.8% of the variance in QOL, ΔR^2^ = 0.148, ΔF (3, 144) = 8.81, *p* < 0.001. In combination, the four predictors explained 19.6% of the variance in QOL, R^2^ = 0.196, adjusted R^2^ = 0.174, F (4, 144) = 8.80, *p* < 0.001, moderate effect (f^2^ = 0.24). As individual predictors, disease severity (sr^2^ = 0.06), MMQ-ability (sr^2^ = 0.03), and MMQ-internal strategy (sr^2^ = 0.03) significantly predicted QOL two years later (see Table 2).

## 4. Discussion

The current study examined the factor structure of the MMQ in an Australian PD community cohort, and the findings support a four-factor structure comprising contentment, ability, internal strategies, and external strategies. The current study then examined whether the four-factor solution predicted QOL at two different time points separated by 2 years. At time 1, three of the four MMQ factors (contentment, ability, and internal strategies) significantly predicted QOL. At time 2 (2 years later), disease severity, ability, and internal strategies significantly predicted QOL. Overall, these findings suggest that perceived memory performance significantly contributes to QOL in those with PD.

A confirmatory factor analysis of the three- and four-factor structures of the MMQ revealed that neither the three- nor the four-factor model demonstrated adequate fit. An EFA was then conducted and resulted in a four-factor solution. Two of the factors, contentment and ability, reflected the original factor structure by Troyer and Rich [12]. The third and fourth factors, internal strategies and external strategies, contained a mixture of items from the original strategies subscale reported by Troyer and Rich [12]. The internal strategies items are related to strategies that use internal mental capacities, such as creating a rhyme to aid remembrance. The external strategies items involve some kind of interaction with the external environment, such as writing something on a calendar. This four-factor structure was reported in previous research with non-English versions of the MMQ [25,26]. All factors were highly internally consistent, supporting the reliability of a four-factor solution.

The four-factor solution reported in the current study has significant implications for future use of this measure, as it reconceptualizes strategy use in a way that facilitates interpretability. Examination of mean scores for the internal and external strategies subscales revealed that the current PD sample used substantially more external strategies than internal strategies. This suggests that those with PD may benefit from memory strategies that involve outward interaction with the environment, such as writing on a calendar, leaving “sticky notes”, or putting something in a prominent place. This is consistent with research indicating that older adults more often use external memory strategies than internal strategies [36,37,38]. A single strategies subscale would therefore not be an accurate representation of strategy use in those with PD. We suggest that findings based on the single strategy subscale of the MMQ should be interpretated with caution until the four-factor model can be verified.

The current study also examined the predictors of QOL in PD. Disease severity, MMQ-contentment, MMQ-ability, and MMQ-internal strategy from time 1 (study entry) significantly predicted QOL at time 2 (2 years later). Higher disease severity was associated with poorer QOL, a finding that is consistent with other PD research [39]. While this is informative, psychological intervention will not reduce the severity of PD symptoms. To develop psychological interventions to improve QOL in PD, an understanding of the contributions made by other predictors, such as metamemory, is required.

Ability significantly predicted QOL, whereby those with higher subjective memory ability at time 1 demonstrated better QOL two years later. This is consistent with previous research in other clinical populations, in which more positive perceptions of memory have been associated with better QOL [6,40]. These findings indicate that interventions to improve QOL in PD should focus on subjective perceptions of memory, as metamemory appears to impact QOL. A person’s subjective perception of their memory ability may impact their QOL more than their objective memory. In those with MCI, subjective memory ability was significantly correlated with QOL, but objective cognitive performance, including memory, was not [41]. This suggests that perceived memory ability may play a more important role than actual memory ability and raises the potential for psychological interventions as a means to manage QOL. As the present study did not assess objective memory performance, future research should examine both objective and subjective memory ability to determine which more strongly predicts QOL in PD. If subjective memory is more predictive of QOL than objective memory performance, there is a clear need to target perceived memory regardless of whether objective memory is impaired.

Internal strategies significantly predicted unique variance in QOL, whereby higher use of internal strategies was associated with poorer QOL two years later. While increased use of memory strategies is typically associated with improved wellbeing [42,43], it may be that the type of memory strategy used (internal vs external strategy) is more important in predicting QOL than overall strategy use. The present study found that higher use of internal strategies was associated with lower QOL, suggesting that internal strategy use is ineffective for those with PD. Conversely, there was no significant relationship between external strategy use and QOL. It is reasonable to suggest that people with PD who rely on internal strategies may find everyday tasks requiring memory much more challenging than those who rely on external strategies. Internal strategies typically involve some use of prospective memory (remembering to remember) and place strain on the available cognitive resources [36]. This is significant when we consider that many people with PD also demonstrate MCI [18]. Unlike internal strategies, external strategies are not as cognitively demanding and typically involve “letting go” of the cognitive load associated with remembering [25,36]. In light of this, people with PD who rely more on external strategies may be less likely to make memory-related mistakes. In addition, some people with PD lack insight into their cognitive functioning, and so do not adapt their strategy use in accord with their cognitive abilities. Raimo et al. [26] and Bouazzaoui et al. [36] suggest that people with good insight into their cognitive functioning might favor external strategies to compensate for cognitive decline. Those people with PD who have reduced insight into their cognitive decline may use more internal strategies. Internal strategies may be less effective than external strategies at reducing the impact of memory decline on QOL. The present study did not explore the efficacy of internal versus external strategy use for the support of memory. It may be that internal strategies are effective in supporting some everyday memory activities and external strategies are effective in supporting others. Future research is required in order to determine the efficacy of internal and external strategies in a range of different situations, as well as how this efficacy impacts QOL.

While the exact mechanism underlying the relationship between metamemory and QOL remains unclear, it may be that some people with PD engage in negative self-appraisals of their memory ability that lead to maladaptive behaviors [44,45]. This may include withdrawing from social situations in which they feel their memory may be challenged [46,47]. The present study revealed that those who rated their memory ability highly were less likely to report using any form of memory strategy (internal or external). These findings were consistent with Csábi et al. [48], who reported that individuals who were more satisfied with their memory ability used both external and internal strategies less frequently. It may be that when a person perceives their memory ability as high (i.e., unimpaired), they do not feel they need to use strategies to complete daily tasks. Those people who rate their memory ability highly are less likely to withdraw from situations that challenge their memory and are more likely to engage in activities that improve QOL [49].

The present findings have significant implications for the development of interventions to improve QOL in PD, whereby metamemory may be a viable avenue through which to intervene for QOL. In stroke, nine one-hour sessions of memory self-efficacy training improved perceptions of memory ability and quality of life, with the improvements maintained over a 12-month period [40]. Improvements were particularly present in individuals under the age of 65, which suggests that younger individuals with clinical conditions may benefit most from memory self-efficacy intervention [40]. This may have important implications for maintaining QOL in individuals with young-onset PD. Future research should examine whether memory self-efficacy training is beneficial to QOL in other clinical populations, such as PD. Additionally, memory self-efficacy has been shown to influence memory strategy use in older adults, whereby those with lower memory self-efficacy were more likely to view memory strategies as difficult to implement compared to individuals with higher memory self-efficacy [50]. This suggests that, if we can intervene to improve perceptions of memory ability, we may also improve memory strategy selection. Given the association between internal strategy use and QOL found in the present study, future research should examine whether interventions that improve effective strategy selection improve QOL in people with PD.

The present findings must be considered in line with a number of limitations. Due to the cut-off score required for informed consent on the MMSE, only individuals who scored above 24 on the MMSE at study entry were included in the present study. Although cognitive function was not included as part of the present study, the high MMSE scores may indicate that the current sample is likely to exhibit very minimal, if any, cognitive impairment [28]. It is reasonable to suggest that this sample of individuals with PD may report better metamemory and QOL compared to individuals with PD who have cognitive impairment. As such, the findings of the present study may not be generalizable to individuals with PD who meet criteria for mild cognitive impairment or PD-Dementia. Future research should examine the influence of cognitive status on the relationship between metamemory and QOL in PD. This would provide valuable insight into whether objective or subjective memory performance is a greater predictor of QOL in PD. Additionally, the current study only included English-speaking individuals. As such, the findings of the present study may not be generalizable to non-English speaking PD populations. Future research should examine the factor structure of the MMQ and the relationship between metamemory and QOL in non-English-speaking PD populations.

## 5. Conclusions

The present study indicates that the relationship between metamemory and QOL that has been reported in other clinical populations extends to PD. Given the degenerative nature of PD, maintaining and promoting good QOL for as long as possible is a significant clinical objective. Understanding the cognitive, psychological, and social predictors of QOL is key to the management of PD. Alongside the training of effective memory strategies for use in everyday situations, interventions that target a person’s perception of their memory ability may be one avenue by which QOL could be improved in PD.

## Figures and Tables

**Table 1:** Frequencies and Demographics (Mean, Standard Deviation) of Sample.

N = 149	M (SD)	Range
Males (number)	98	-
Age at diagnosis	59.07 (10.89)	25–84
Age at participation	65.78 (9.25)	39–85
Hoehn and Yahr	1.79 (0.70)	1–5
MMSE	27.84 (1.79)	22–30
Married	117	-

Note. MMSE = Mini Mental State Examination. Participants who scored <24 at entry were excluded.

**Table 2:** Hierarchical multiple regression analysis predicting quality of life from disease severity and metamemory components.

Variable	*B* [95% CI]	ß	*sr* ^2^
Step 1			
Disease Severity	4.38 [1.23–7.52] **	0.22	0.05
Step 2			
Disease Severity	5.01 [2.07–7.95] ***	0.25	0.06
MMQ-Contentment	−0.034 [−0.25–0.18]	−0.03	0.00
MMQ-Ability	−0.32 [−0.56–−0.07] *	−0.27	0.03
MMQ-Internal Strategies	0.47 [0.059–0.87] *	0.18	0.03

Note. CI = confidence interval. MMQ = Multifactorial Memory Questionnaire. * *p* < 0.05. ** *p* < 0.01. *** *p* < 0.001.

## Data Availability

Data are available upon reasonable request from the corresponding author. The data are not publicly available due to the nature of the overarching project.

## Abbreviations

The following abbreviations are used in this manuscript:

PD	Parkinson’s disease
MMQ	Multifactorial Memory Questionnaire
QOL	Quality of life
MCI	Mild cognitive impairment
H&Y	Hoehn and Yahr Scale
PDQ-39	Parkinson’s Disease Questionnaire-39
MMSE	Mini Mental State Examination
EFA	Exploratory factor analysis
